# Dynamics and function of CXCR4 in formation of the granule cell layer during hippocampal development

**DOI:** 10.1038/s41598-017-05738-7

**Published:** 2017-07-17

**Authors:** Yuka Mimura-Yamamoto, Hiroshi Shinohara, Taichi Kashiwagi, Toru Sato, Seiji Shioda, Tatsunori Seki

**Affiliations:** 1 Department of Histology and Neuroanatomy, Tokyo Medical University, Tokyo, 160-8402 Japan; 2 Institute for advanced Bioscience Research, Hoshi University, Tokyo, 142-8501 Japan

## Abstract

In the developing hippocampus, granule cell progenitors (GCPs) arising in the ventricular zone (VZ) migrate to the subpial region, and form the granule cell layer (GCL) of the dentate gyrus (DG). To understand the mechanism of GCL formation, we investigated the dynamics and function of CXCR4 which is expressed by the GCPs and is a receptor of the CXCL12 chemokine secreted by cells surrounding the DG. In the VZ, CXCR4 was expressed on the plasma membrane of the GCPs. During their migration and in the DG, CXCR4 was internalized and accumulated as puncta close to the centrosomes, Golgi apparatus, and lysosomes. Phosphatase analysis suggested that both phosphorylated and dephosphorylated CXCR4 exist on the plasma membrane, whereas CXCR4 in intracellular puncta was mainly dephosphorylated. Intraventricular administration of the CXCR4 antagonist AMD3100 resulted in the disappearance of CXCR4 expression from the intracellular puncta, and its appearance on the plasma membranes. Furthermore, AMD3100 treatment resulted in precocious differentiation, delayed migration, and ectopic GCPs. Taken together, these results suggest that during the development and migration of GCPs, CXCR4 on the plasma membrane is phosphorylated, internalized, sorted to the centrosomes, Golgi apparatus, and lysosomes, and functionally regulates GCP differentiation, migration and positioning.

## Introduction

In the dentate gyrus (DG) of the hippocampus, granule cells are continuously generated throughout the life of mammals by granule cell progenitors (GCPs) that express glial fibrillary acidic protein (GFAP)^1–6^. Postnatal neurogenesis has been reported to be associated with various hippocampal functions, such as memory and learning^3, 4, 7, 8^, as well as brain diseases, including epilepsy, ischemia, and mental diseases^9, 10^. To understand the mechanisms of this persistent neurogenesis, comprehensive analysis of the neurogenesis of dentate granule cells, from embryonic to adult stages is required.

During the embryonic stages, GCPs first appear in the ventricular zone (VZ) of the ventral region of the medial pallium, migrate to the subpial region, and form the anlage of the DG^11–15^. As development proceeds, the migrating GCPs form the dentate migratory stream (DMS) along the suprafimbrial and subpial regions of the fimbrio-dentate junction, where the GCPs continue to proliferate and produce granule cell precursors. Simultaneously, Cajal-Retzius (CR) cells accumulate in a region surrounding the hippocampal fissure and subpial region to delineate the C-shaped border of the DG^16–18^.

Recently, molecular biological analyses have demonstrated that the production and migration of GCPs are regulated by various secreted proteins, such as CXCL12, reelin, Wnt, and BMP^14, 15, 19–22^. *In situ* hybridization analysis demonstrated that in the developing hippocampus, CXCL12 is expressed in the CR cells, and its receptor, C-X-C chemokine receptor 4 (CXCR4) is expressed in GCPs^18, 19, 20, 23^. Studies using CXCR4 or CXCL12 knockout mice have suggested that development of the granule cell layer (GCL) is regulated by CXCL12/CXCR4 signaling^14, 15, 19, 20^. CXCL12 is a member of the C-X-C subfamily of chemokines (also known as stromal cell- derived factor-1, SDF-1) and its receptor CXCR4 belongs to the G-protein coupled receptor family. CXCL12/CXCR4 signaling is reported to be involved in various biological processes, including the immune response, hematopoiesis, cardiogenesis, angiogenesis, neurogenesis, germ cell migration, and metastasis of cancer cells^24–27^. In these processes, it has been shown that phosphorylation and intracellular trafficking of CXCL12/CXCR4 are essential for regulating the proliferation, differentiation, and migration of stem/progenitor cells. However, in the nervous system, the precise dynamics and roles of CXCR4 in the production, migration, and differentiation of neural progenitors remain unclear.

Therefore, in this study, we examined the dynamics of the CXCR4 protein in migrating GCPs using *Gfap*-GFP mice that enable the visualization of GFAP-expressing GCPs and their immediate neural progeny^13^. We also used the novel antibody UMB-2 that specifically recognizes non-phosphorylated CXCR4^28^, and lambda protein phosphatase (λPP) to identify total CXCR4 (phosphorylated and non-phosphorylated CXCR4). Furthermore, to clarify the role of CXCL12/CXCR4 signaling, the CXCR4 antagonist AMD3100 was intraventricularly administered to the mice. AMD3100 is able to selectively inhibit the binding of CXCL12 to CXCR4 which is required for intracellular signaling, phosphorylation of the C-terminal portion of the CXCR4 and internalization of CXCR4^29–31^. Our present results demonstrate that CXCR4 is internalized and dephosphorylated during the migration of GCPs, and functions in the differentiation and migration of GCPs.

## Results

### Expression pattern of CXCR4 during development of the dentate gyrus

To clarify the expression pattern of the CXCR4 protein in the developing cortex, including the hippocampal region, we performed immunohistochemical analysis using the UMB-2 antibody, which specifically recognizes non-phosphorylated CXCR4, because a comparative study of commercially available CXCR4 antibodies demonstrated that the UMB-2 antibody can efficiently detect true CXCR4 plasma membrane receptors in contrast to other antibodies^28, 32^. In fact, although we tested other commercially available antibodies that are documented to recognize phosphorylated CXCR4, they did not yield reliable and specific staining results in immunohistochemistry (data not shown).

**Figure 1:**
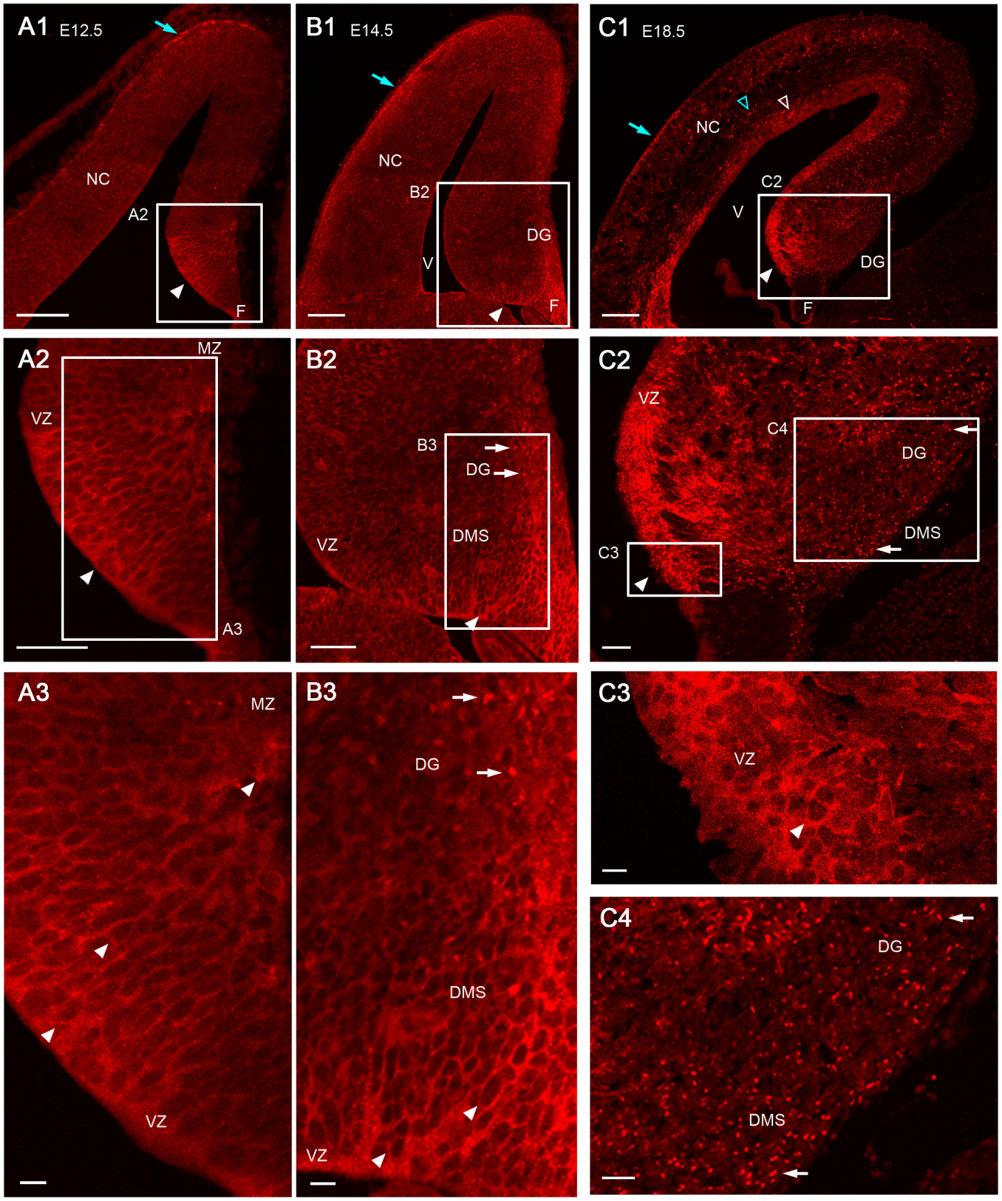
Developmental changes of CXCR4 expression in the cerebral hemisphere, including the neocortex and hippocampus at E12.5 (**A**), E14.5 (**B**), and E18.5 (**C**). The boxed regions in **A1**, **B1**, and **C1** are enlarged in **A2**, **B2**, and **C2**, respectively. The boxed regions in **A2**, **B2**, and **C2** are enlarged in **A3**, **B3**, and **C3** and 4, respectively. (**A**) At E12.5, strong CXCR4 expression is restricted to the anlage of the dentate gyrus (arrowheads, **A1, 2**), and is found on the plasma membrane of cells located from the ventricular zone (VZ) to the marginal zone (MZ, **A3**). (**B**) At E14.5, strong CXCR4 expression is present in the VZ around the dentate notch (arrowheads, **B1, 2**). CXCR4 expression is present on the plasma membrane of cells from the VZ to the dentate migratory stream (DMS) including the suprafimbrial region and subpial regions of the fimbryo-dentate junction (arrowheads, **B3**), whereas a small number of CXCR4-positive puncta are observed in the dentate gyrus (DG, white arrows, **B2**, 3). (**C**) At E18.5, strong CXCR4 expression is detected in the VZ around the dentate notch (white arrowhead, C1) where CXCR4 expression is present in the plasma membrane of cells (white arrowheads, **C2**, 3). However, many CXCR4-positive puncta are distributed broadly from the DMS to the DG (white arrows, **C2**, 4). In the neocortex, CXCR4 is expressed in the marginal zone (blue arrows, **A1**, **B1**, **C1**), intermediate zone (blue open arrowhead, **C1**) and VZ (white open arrowhead, **C1**) in agreement with a previous study^33^. F, fimbria; NC, neocortex; V, ventricle. Scale bars = 100 µm in **A1** and **B1**; 200 µm in **C1**; 50 µm in **A2–C2**.

In sections of the cerebral hemisphere, CXCR4 expression was observed in the marginal zone (MZ, Fig. 1A1, B1, and C1), intermediate zone, and VZ (Fig. 1C1), in agreement with previous studies^33^, suggesting that the UMB-2 antibody is reliable for use in immunohistochemistry.

At embryonic day 12.5 (E12.5), strong CXCR4 expression was found in the most ventral region of the medial pallium corresponding to the hippocampal anlage (Fig. 1A1). CXCR4 expression was observed in the plasma membrane of cells located from the VZ to the MZ (Fig. 1A2). In the hippocampus at E14.5 (Fig. 1B1), CXCR4 expression on the plasma membrane was detected in the VZ and DMS, including the suprafimbrial regions and the fimbrio-dentate junction (Fig. 1B), and a small number of CXCR4-positive puncta appeared in the developing DG (Fig. 1B). This punctate CXCR4 expression spread from the DG to the DMS at E18.5, whereas CXCR4 expression on the plasma membrane was absent in the DMS (Fig. 1C1). These results suggest the possibility that the distribution pattern of CXCR4 within a cell is associated with the migration and development of GCPs. The distribution pattern of cells expressing CXCR4 that we observed is in good agreement with that previously demonstrated for CXCR4 mRNA by *in situ* hybridization analysis^19, 20, 23^.

### Changes in CXCR4 expression pattern during GCP migration

**Figure 2:**
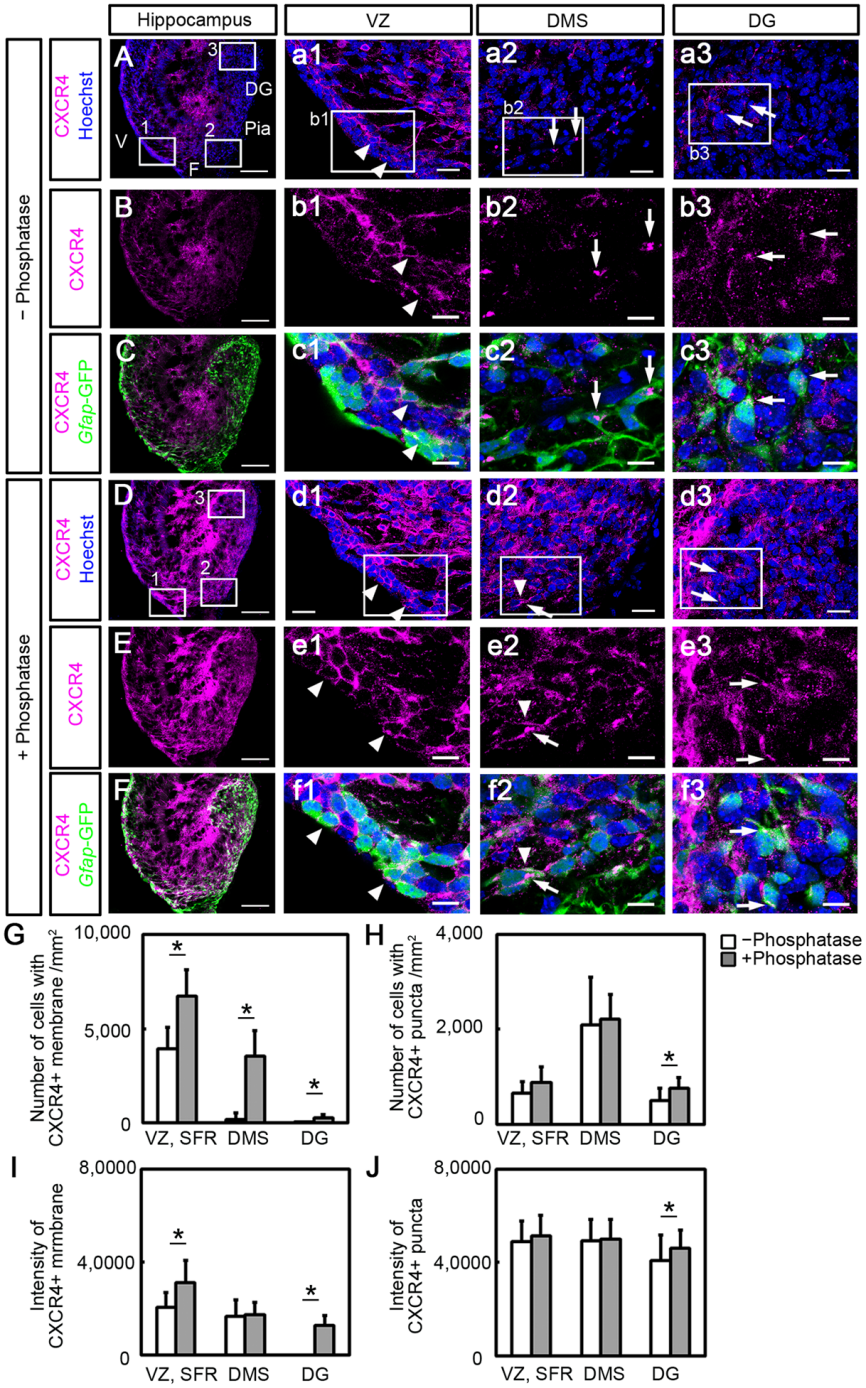
Non-phosphorylated (**A–C**) and total (**D–F**) CXCR4 expression in the hippocampus of *Gfap*-GFP mice at E18.5. Non-phosphorylated CXCR4 was detected by the UMB2 antibody, which specifically recognizes the non-phosphorylated C-terminal domain of CXCR4. To detect total CXCR4 (both the phosphorylated and non-phosphorylated forms), sections of the hippocampus were treated with lambda protein phosphatase (λPP). Immunostaining with or without λPP in adjacent sections was performed simultaneously in the same condition. (**A–C**) Detection of non-phosphorylated CXCR4. The boxed regions in A are enlarged in a1, a2 and a3, as indicated. The boxed regions in a1–3 are enlarged in b1–3 and c1–3, respectively. In the ventricular zone (VZ), CXCR4 is present in the plasma membrane of *Gfap*-GFP-expressing cells (arrowheads, a1, b1, and c1). In the dentate migratory stream (DMS) and dentate gyrus (DG), CXCR4 accumulates as puncta at the base of processes of *Gfap*-GFP-expressing cells (arrows, b2, b3, c2, and c3). (**D–F**) Total CXCR4 expression. The boxed regions in D are enlarged in d1, d2, and d3, as indicated. The boxed regions in d1–3 are enlarged in e1–3 and f1–3, respectively. Quantitative analysis of the number of cells with CXCR4+ plasma membranes and intracellular puncta, and immunoreactive intensities of CXCR4 in the membrane and puncta are shown in (**G, H**) and (**I, J**), respectively. Note that the immunoreactivity for total CXCR4 (**E**) is much higher than for non-phosphorylated CXCR4 (**B**). Note that the numbers of cells with CXCR4 on the plasma membrane in the phosphatase-treated group (+phosphatase) is increased in the VZ+ suprafimbrial region (SFR), DMS, and DG, compared with the phosphatase non-treated (−phosphatase) group (b1–b3, e1–e3 and **G**, *P < 0.05, n = 9, 9 sections in 3 embryos, Student t-test), and that the intensity of CXCR4 on the plasma membrane in the +phosphatase group is increased in the VZ-SFR and DG (b1, b3, e1, e3, I, *P < 0.05, n = 9, 9 sections in 3 embryos, Student t-test). F, fimbria; V, ventricle. Scale bars = 100 µm in **A, B, C, D, E**, and **F**; 20 µm in a1–3, and d1–3; 10 µm in b1–3, c1–3, e1–3, and f1–3.

To clarify whether CXCR4 is expressed by GCPs, we used *Gfap-*GFP transgenic mice, because we previously demonstrated that GCPs express GFAP from the beginning of dentate development, and *Gfap*-GFP expressing cells accurately represent the GCPs and their immediate neural progeny^13^. In the VZ at E18.5, CXCR4 was expressed on the plasma membrane of *Gfap-*GFP-positive (*Gfap*-GFP+) cells (Fig. 2A,B,C,a1,b1, and C1). In the DMS and DG, CXCR4 immunoreactivity was observed as punctate structures at the base of processes of *Gfap*-GFP+ fusiform-shaped cells (Fig. 2 a2,a3,b2,b3,c2, and c3). However, we also found punctate CXCR4 immunoreactivity in *Gfap*-GFP-negative (*Gfap*-GFP−) areas where *Gfap*-GFP-negative migrating neuronal precursors could be present. To confirm this, we labeled migrating cells by *in utero* electroporation, in which the *pCAGGS*-BFP plasmid was electroporated into the ventricular surface of the hippocampal region. We found CXCR4 puncta in BFP+/*Gfap*-GFP− cells with a nucleus positive for NeuroD, an immature neuronal marker, suggesting the existence of punctate CXCR4 in neuronal precursors in which *Gfap* promoter activity is downregulated (Supplemental Fig. 1). Additionally, to demonstrate the early distribution pattern of CXCR4 in *Gfap*-GFP cells, we analyzed the prospective hippocampus at E14.5 (Supplemental Fig. 2). The distribution pattern of CXCR4 in the E14.5 prospective hippocampus was similar to that in the E18.5 hippocampus. In the VZ, CXCR4 was expressed on the plasma membrane of *Gfap*-GFP cells. In the DMS and prospective dentate gyrus, CXCR4 was found as puncta in the *Gfap*-GFP cells. These results suggest that during migration of granule progenitors, CXCR4 distribution is altered from the plasma membrane to intracellular puncta.

To detect total CXCR4 (phosphorylated and non-phosphorylated CXCR4), sections of the hippocampus were treated with λPP. This treatment greatly enhanced CXCR4 immunoreactivity in the hippocampus (Fig. 2D–F), and appeared to increase the number of CXCR4+ cells. In this experiment, the increase in CXCR4 immunoreactivity in sections with λPP treatment indicates the presence of phosphorylated CXCR4. Furthermore, the increase in the number of CXCR4+ cells after λPP treatment indicates the existence of cells with only phosphorylated CXCR4. Hence, we counted the number of cells expressing CXCR4 on the plasma membrane and in puncta in sections with (+PP) or without (−PP) λPP treatment. The number of cells expressing CXCR4 on the plasma membrane was increased in the VZ-suprafimbrial region (SFR, Fig. 2d1–f1, and G; −PP, 3929 ± 653 cells/mm^2^ vs. +PP, 6744 ± 1057 cells/mm^2^, P < 0.05, n = 9, 9 sections in 3 embryos, Student t-test), DMS (Fig. 2 d 2–f2, and G; −PP, 193 ± 365 cells/mm^2^ vs. +PP, 3561 ± 1315 cells/mm^2^, P < 0.05, n = 9, 9 sections in 3 embryos) and DG (Fig. 2G; −PP, 0 ± 0 cells/mm^2^ vs. +PP, 286 ± 145 cells/mm^2^, P < 0.05, n = 9, 9 sections in 3 embryos), compared with those in sections without phosphatase treatment (Fig. 2 a 1–c1, a2–c2). On the other hand, there was no change in the number of CXCR4 +puncta in the VZ-SFR (Fig. 2H; −PP, 662 ± 239 cells/mm^2^ vs. + PP, 893 ± 347 cells/mm^2^, P = 0.118, n = 9, 9 sections in 3 embryos, Student t-test) and DMS (Fig. 2H; −PP, 2098 ± 1003 cells/mm^2^ vs. + PP, 2221 ± 519 cells/mm^2^, P = 0.761, n = 9, 9 sections in 3 embryos), although there was a slight increase in the DG (Fig. 2H; −PP, 511 ± 252 cells/mm^2^ vs. + PP, 769 ± 226 cells/mm^2^, P < 0.05, n = 9, 9 sections in 3 embryos) between before and after phosphatase treatment (Fig. 2H). To analyze the phosphorylation state of CXCR4 on the plasma membrane and in intracellular puncta, we measured the intensity of CXCR4 immunofluorescence. In this experiment, immunostaining with or without λPP was performed simultaneously in adjacent sections under the same conditions. On the plasma membrane of cells, the staining intensity increased in the VZ-SFR (Fig. 2I; −PP, 20754 ± 6375 vs. +PP, 31367 ± 9475, P < 0.05, n = 9, 9 sections in 3 embryos, Student t-test) and the DG (Fig. 2I; −PP, 0 ± 0 vs. +PP, 12835 ± 4433, P < 0.05, n = 9, 9 sections in 3 embryos), but not in the DMS (Fig. 2I; −PP, 16669 ± 7284 vs. +PP, 17439 ± 5497, P = 0.693, n = 9, 9 sections in 3 embryos). On the intracellular puncta of cells, the staining intensity increased in the DG (Fig. 2J; −PP, 40960 ± 10876 vs. +PP, 45963 ± 7931, P < 0.05, n = 9, 9 sections in 3 embryos, Student t-test), but not in the VZ-SFR (Fig. 2J; -PP, 48929 ± 8965 vs. +PP, 51431 ± 8858, P = 0.092, n = 9, 9 sections in 3 embryos) and DMS (Fig. 2J; −PP, 49174 ± 9141 vs. +PP, 49877 ± 8539, P = 0.608, n = 9, 9 sections in 3 embryos), suggesting that both phosphorylated and dephosphorylated CXCR4 exist on the plasma membrane in the VZ-SFR, whereas the dephosphorylated form is mainly present in intracellular puncta of cells in the VZ-SFR and DMS. It was also noted that although the intensity of CXCR4 immunoreactivity on the plasma membrane of cells in the DG increased, the number of cells with CXCR4 on the plasma membrane was very low. Additionally, to know the extent of phosphorylation of CXCR4, the percentage of cells with dephosphorylated CXCR4 to cells with total (both phosphorylated and dephosphorylatedtotal) CXCR4 was calculated, and the percentage of cells with phosphorylated CXCR4 membrane was obtained by subtraction of the percentage of cells with dephosphorylated CXCR4 on the plasma membrane from cells with total CXCR4 on the plasma membrane (Supplemental Fig. 3). The percentage of cells with dephosphorylated CXCR4 on the plasma membrane decreased from the VZ to the DG (VZ + SFR, 62.9 ± 27.4%, DMS, 3.7 ± 5.7%, DG, 0 ± 0%; VZ + SFR vs. DMS, P < 0.01, VZ + SFR vs. DG, P < 0.01, Tukey-Kramer test), whereas the percentage of cells with phosphorylated CXCR4 on the plasma membrane gradually increased from the VZ to the DG (VZ + SFR, 37.1 ± 27.4%, DMS, 96.3 ± 5.7%, DG, 100 ± 0%; VZ + SFR vs. DMS, P < 0.01, VZ + SFR vs. DG, P < 0.01, Tukey-Kramer test; Supplemental Fig. 3A). On the other hand, there was no change in the percentage of cells with phosphorylated CXCR4+ puncta among the VZ + SFR, DMS, and DG (VZ + SFR, 82.9 ± 38.8%, DMS, 96.7 ± 44%, DG, 71.8 ± 33.5%, P = 0.453, one-way ANOVA; Supplemental Fig. 3B). The results suggest that CXCR4 on the plasma membrane is gradually phosphorylated as GCPs migrate from the VZ to the DG through the DMS, and that the CXCR4+ puncta are mainly dephosphorylated.

### Intracellular distribution of CXCR4+ puncta in GCPs

**Figure 3:**
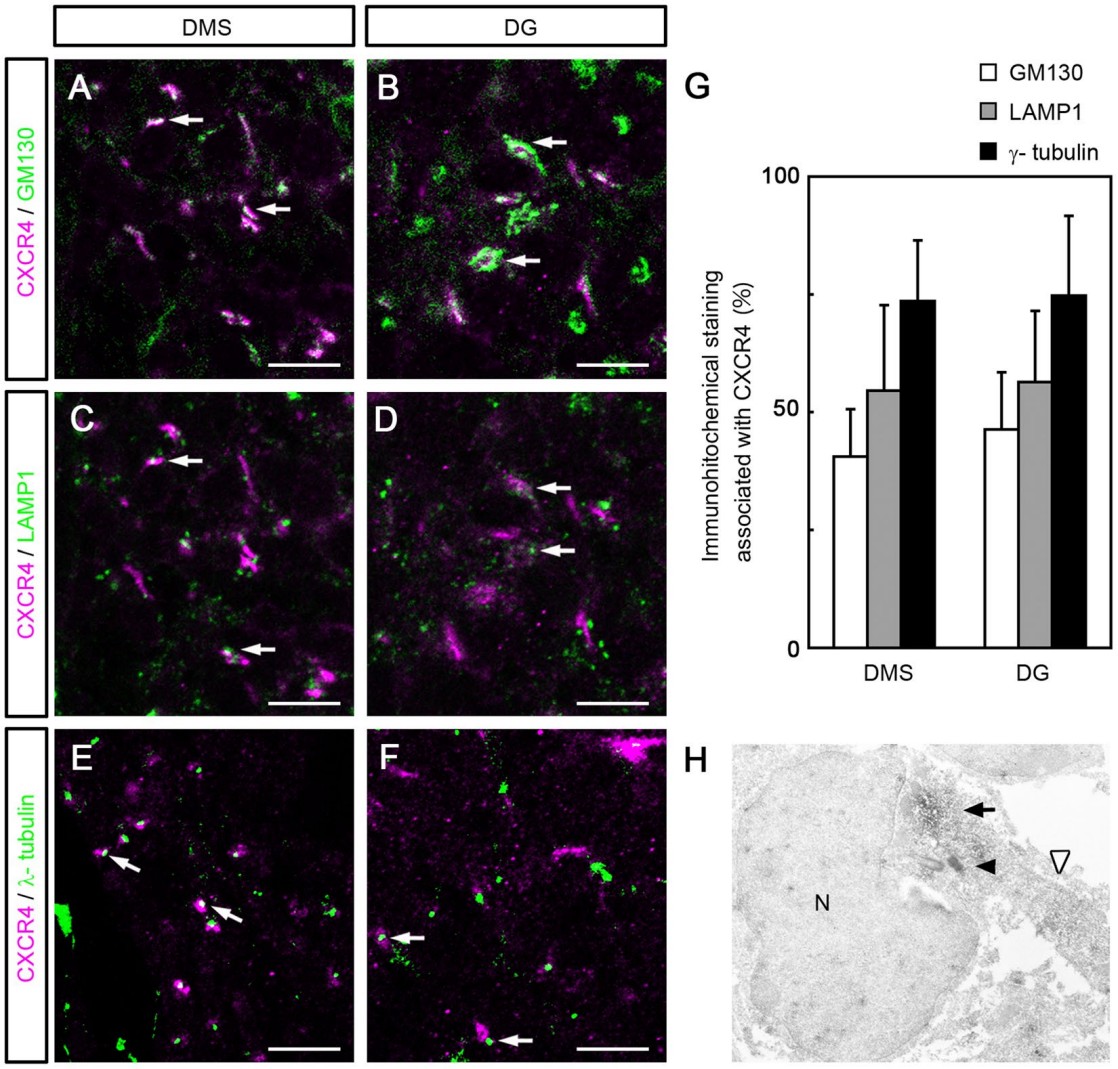
Association of CXCR4-positive aggregates with the Golgi apparatus marker GM130 (**A** and **B**), the lysosome marker LAMP1 (**C** and **D**), and the centrosome marker γ-tubulin (**E** and **F**), in the dentate migratory stream (DMS, **A**,**C**, and **E**) and dentate gyrus (DG, **B**,**D**, and **F**) of E17.5 mice. A fraction of the CXCR4+ aggregates contact or overlap with GM130, LAMP1, and γ-tubulin immunoreactivity (arrows). (**G**) Quantitative analysis of the proportion of CXCR4+ puncta associated with immunoreactivities of GM130, LAMP1, and γ-tubulin among total CXCR4+ puncta in the DMS and DG (n = 3, 9 sections in 3 embryos). (**H**) Immunoelectron microscopy showing CXCR4 immunoreactivity (black arrow) localized near the centrosome (black arrowhead) at the base of a process (white arrowhead). Scale bar = 10 µm in **A–F**.

Because in the immune system, CXCR4 is internalized and sorted to organelles such as the lysosome^24^, we next investigated whether CXCR4+ puncta in the DMS and DG are associated with any organelles. To detect the Golgi apparatus, lysosome and centrosome, we used antibodies for GM130, LAMP1, and γ-tubulin, respectively. In the DMS, 40.5%, 54.6% and 73.6% of CXCR4-positive puncta were in contact with structures immunoreactive for GM130, LAMP1 and γ-tubulin, respectively (Fig. 3A,C,E, and G). In the DG, 46.3%, 56.4%, and 74.7% of CXCR4-positive puncta were in contact with GM130, LAMP1, and γ-tubulin-immunoreactive structures, respectively (Fig. 3B,D,F, and G, n = 3, 9 sections in 3 embryos). Furthermore, immunoelectron microscopy demonstrated that CXCR4 immunoreactivity is often localized near the centrosome at the base of a process (Fig. 3H). These results suggest the possibility that CXCR4 may be sorted to the Golgi apparatus, lysosomes, and centrosomes, and may hence function with these organelles.

### Inhibition of CXCL12/CXCR4 signaling alters the intracellular localization of CXCR4

**Figure 4:**
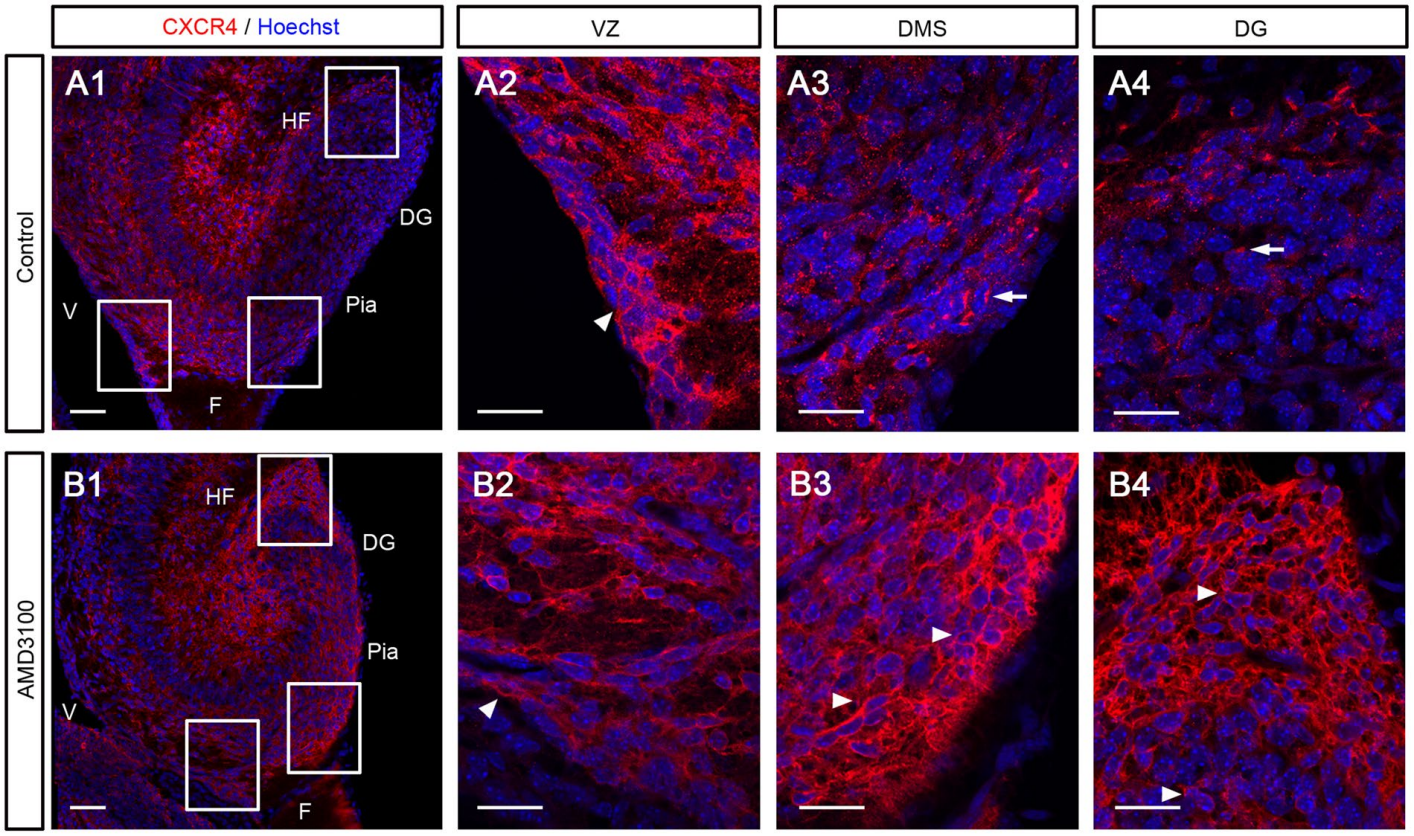
The CXCR4 antagonist AMD3100 alters CXCR4 expression patterns in the developing hippocampus. AMD3100 or vehicle (control) was injected into the telencephalic lateral ventricle of E15.5 embryos *in utero*, and embryos were left to develop until E18.5 before sampling. The boxed regions in **A1** and **B1** are enlarged in **A2–4** and **B2–4**, respectively. In control mice, CXCR4 expression was observed in the plasma membrane of cells in the ventricular zone (VZ, **A1**, 2, arrowhead), and in the puncta of cells in the DMS and DG (**A1**, 3, 4, arrows). However, in AMD3100-treated mice, all CXCR4 expression was observed in the plasma membrane in the VZ, DMS, and DG (**B1–4**, arrowheads). DG, dentate gyrus; F, fimbria; HF, hippocampal fissure; V, ventricle. Scale bars = 50 µm in **A1** and **B1**; 20 µm in **A2–4** and **B2–4**.

We next investigated whether the formation of CXCR4-positive puncta in the DMS and DG is regulated by CXCL12/CXCR4 signaling, because it is widely accepted that CXCR4 is internalized by CXCL12 stimulation^34–38^. To this end, the CXCR4 antagonist AMD3100^29–31^ was injected into the telencephalic lateral ventricle of E15.5 embryos *in utero*, and embryos were left to develop until E18.5. In the whole of the hippocampus, no CXCR4+ puncta as seen in control were detected in the AMD3100-treated mice, and instead all CXCR4 staining was found in the plasma membrane (Fig. 4). Dephosphorylation by λPP did not significantly alter the number of cells with CXCR4 on their plasma membrane in the VZ-SFR (-PP, 2290 ± 2384 cells vs. +PP, 4320 ± 1428 cells, P = 0.134, n = 6, 6 sections in 6 embryos, Student t-test) and DMS (-PP, 5408 ± 2706 cells vs. +PP 8912 ± 3604 cells, P = 0.113, n = 6, 6 sections in 6 embryos, Student t-test) in AMD3100-treated embryos, suggesting that the majority of migrating cells have dephosphorylated CXCR4 in AMD3100-treated embryos, probably because of the absence of CXCL12 stimulation (Supplemental Fig. 4). In the VZ, the expression pattern of CXCR4 was basically similar between control and AMD3100-treated mice (Figs 4 A 1, 2, B1, and B2). In the DMS and DG, strong CXCR4 expression was observed on the plasma membrane (Fig. 4A3, A4, B3, and B4). Additionally, CXCR4 cells appeared to be accumulated around the hippocampal fissure. These results suggested that CXCL12/CXCR4 signaling controls the formation of CXCR4+ puncta.

### Inhibition of CXCL12/CXCR4 signaling induces precocious neural differentiation in the VZ

**Figure 5:**
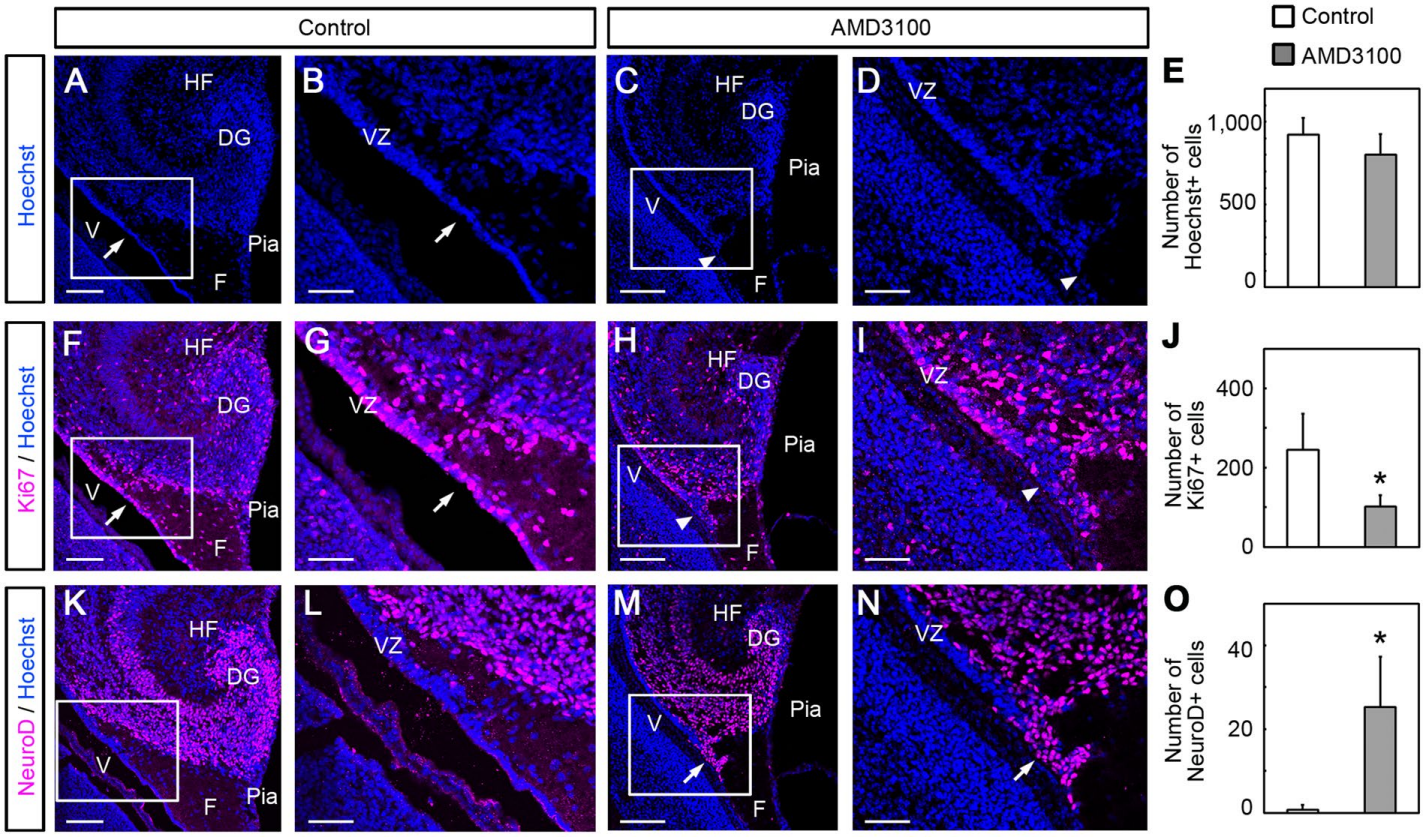
Precocious differentiation of GCPs in the VZ induced by the CXCR4 antagonist AMD3100. AMD3100 or vehicle (control) was injected into the telencephalic lateral ventricle of E15.5 embryos *in utero*, and the embryos were left to develop until E18.5 before sampling. Nuclear distribution (**A–D**) and immunoreactivities for Ki67 (**F–I**) and NeuroD (**K–O**) in the VZ are shown. The boxed regions in **A, C, F, H, K**, and **M** are enlarged in **B, D, G, I, L**, and **N**, respectively. In the control, numerous Ki67 positive cells, which represent proliferating cells, are observed in the VZ (arrows in **F** and **G**), whereas such cells are sparsely observed in the AMD3100-treated embryos (arrowheads in **H** and **I**). The number of Ki67 positive cells is decreased in the AMD3100-treated group (**J**, *P < 0.05, Student t-test). On the other hand, NeuroD positive cells were rarely observed in the VZ of control embryos (**K** and **L**), whereas they were frequently observed in the AMD3100-treated embryos (arrows in **M** and **N**). The number of NeuroD positive cells increased in the AMD3100-treated group (**O**, *P < 0.05, Student t-test). These results suggested that dentate progenitors are precociously differentiated in the VZ by the inhibition of CXCR4 signaling. Ten sections were counted from each of 3 (control) or 4 (AMD3100-treated) animals. DG, dentate gyrus; **F**, fimbria; HF, hippocampal fissure; V, ventricle. Scale bars = 100 µm in **A, C, F, H, K**, and **M**; 50 µm in **B, D, G, I, L**, and **N**.

To understand the properties of cells in the VZ after AMD3100 administration, we examined the expression of the proliferating cell marker Ki67, and the neural progenitor marker NeuroD. Initially we counted the number of Hoechst-labeled nuclei in the VZ at a distance of 250 μm dorsal from the dentate notch, because it corresponds approximately to the GCP-generating region. In the present study, we will refer to this as the GCP-generating ventricular zone (GCP-VZ). In the GCP-VZ (Fig. 5A–E), we found no differences (P = 0.231, Student t-test) in the number of Hoechst-labeled cells between the control (921 ± 101 cells in the GCP-VZ, n = 3, 10 sections from each of 3 embryos) and AMD3100-treated embryos (801 ± 125 cells in the GCP-VZ, n = 4, 10 sections from each of 4 embryos). Immunohistochemical analysis showed that in the control, Ki67+ cells were densely packed in the VZ, but in the AMD3100-treated mice, they were sparsely distributed (Fig. 5F–I). The average number of Ki67+ cells in the GCP-VZ were significantly decreased (P < 0.05, Student t-test) in AMD3100-treated mice (103 ± 29 cells in the GCP-VZ, n = 3, 30 sections in 3 embryos) compared with control mice (245 ± 91 cells in the GCP-VZ, n = 4, 40 sections in 4 embryos, Fig. 5J). In the control mice, no NeuroD+ cells were observed in the GCP-VZ (1 ± 1 cells in the GCP-VZ, n = 3, 30 sections in 3 embryos), but the number of NeuroD+ cells significantly increased (P < 0.05, Student t-test) in the AMD3100-treated mice (25 ± 12 cells in the GCP-VZ, n = 4, 40 sections in 4 embryos, Fig. 5K–O). These results suggest that inhibition of CXCL12/CXCR4 signaling leads to the precocious differentiation of ventricular progenitor cells.

### Inhibition of CXCL12/CXCR4 signaling induces delayed migration and precocious differentiation of GCPs

**Figure 6:**
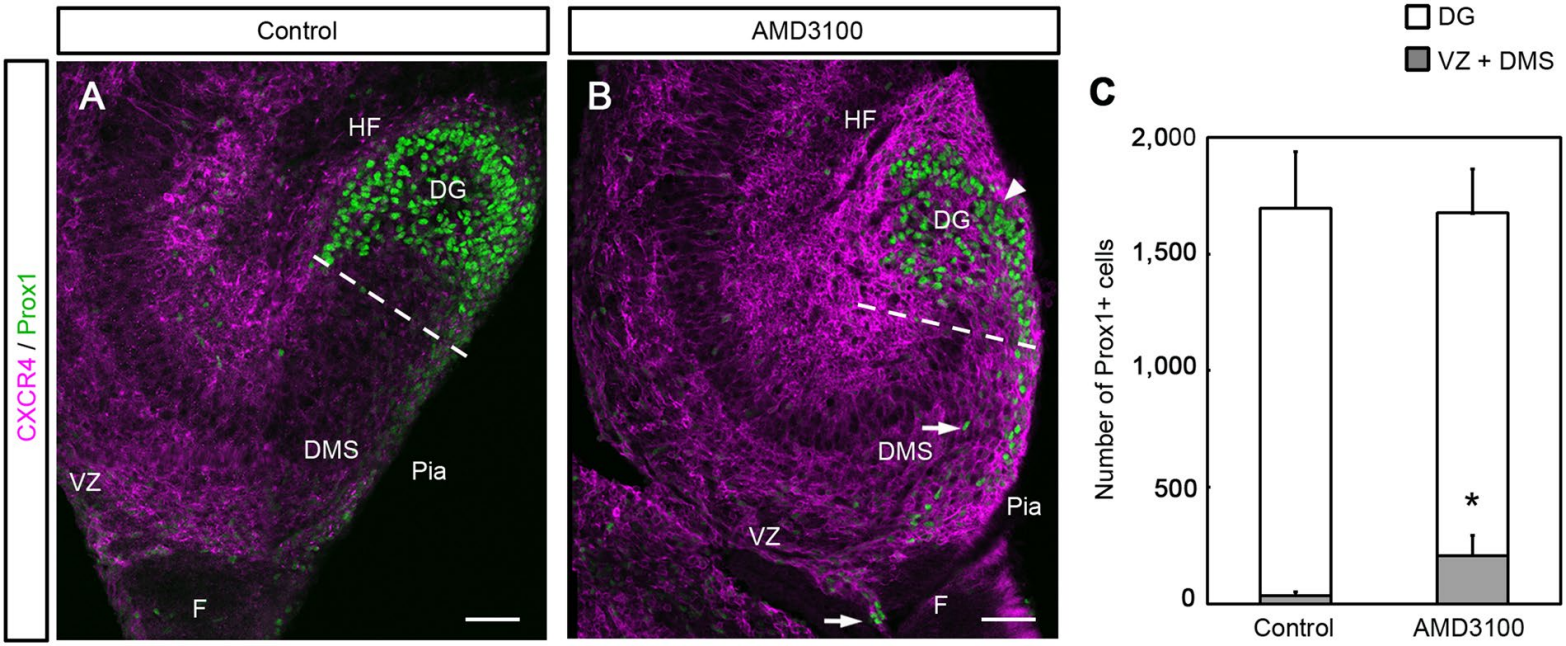
Altered distribution pattern of Prox1-positive granule cell precursors induced by the CXCR4 antagonist AMD3100. AMD3100 or vehicle (control) was injected into the telencephalic lateral ventricle of E15.5 embryos *in utero*, and the embryos were left to develop until E18.5 before sampling. (**A**,**B**) Immunoreactivity for CXCR4 and Prox1, a marker for granule cells in the hippocampus at E18.5. (**A**) In the control, cells strongly positive for Prox1 were mainly localized in the dentate gyrus (DG). (**B**) In AMD3100-treated mice, cells strongly positive for Prox1 were observed not only in the DG (arrowhead), but also in the ventricular zone (VZ) and dentate migratory stream (DMS, arrows). (**C**) The number of Prox1-positive cells in the DG, and in the VZ and DMS. The number of Prox1-positive cells in the VZ and DMS is significantly higher in the AMD3100-treated mice than in control mice (*P < 0.05, Student’s t test). No significant difference was observed in the total number of Prox1-positive cells between the two groups. Ten sections were counted from each of 5 (control) or 6 (AMD3100-treated) animals. F, fimbria; HF, hippocampal fissure; V, ventricle. Scale bar = 50 µm in **A** and **B**.

To investigate whether CXCL12/CXCR4 signaling regulates the final differentiation of granule cells, we analyzed the expression of the granule cell marker Prox1. AMD3100 was injected into the telencephalic lateral ventricle of E15.5 embryos *in utero*, and embryos were left to develop until E18.5. In control embryos, cells strongly positive for Prox1 were mainly localized in the DG (Fig. 6A), but in AMD3100 treated-embryos, cells with strong ectopic expression of Prox1 were detected in the VZ and DMS (Fig. 6B). The average number of cells with strong Prox1 staining in the VZ and DMS significantly increased (P < 0.05; Student t-test) in the AMD3100-treated mice (207 ± 87 cells, n = 6, 10 sections from each of 6 embryos) compared with control mice (37 ± 14 cells, n = 5, 10 sections from each of 5 embryos, Fig. 6C). However, no difference was observed in the total number of Prox1-positive cells in the VZ + DMS and DG between the two conditions (Fig. 6C; control, 1,696 ± 235 cells vs. AMD3100, 1,675 ± 156 cells, P = 0.875, n = 5 and 6, 10 sections from each of 5 or 6 embryos, respectively, Student t-test), suggesting that inhibition of CXCL12/CXCR4 signaling does not alter the production of granule cells itself. These results suggest that CXCL12/CXCR4 signaling affects the development of granule cells by regulating the migration and maturation of GCPs.

### Inhibition of CXCL12/CXCR4 signaling results in ectopic *Gfap*-GFP+ cells surrounding the hippocampal fissure

**Figure 7:**
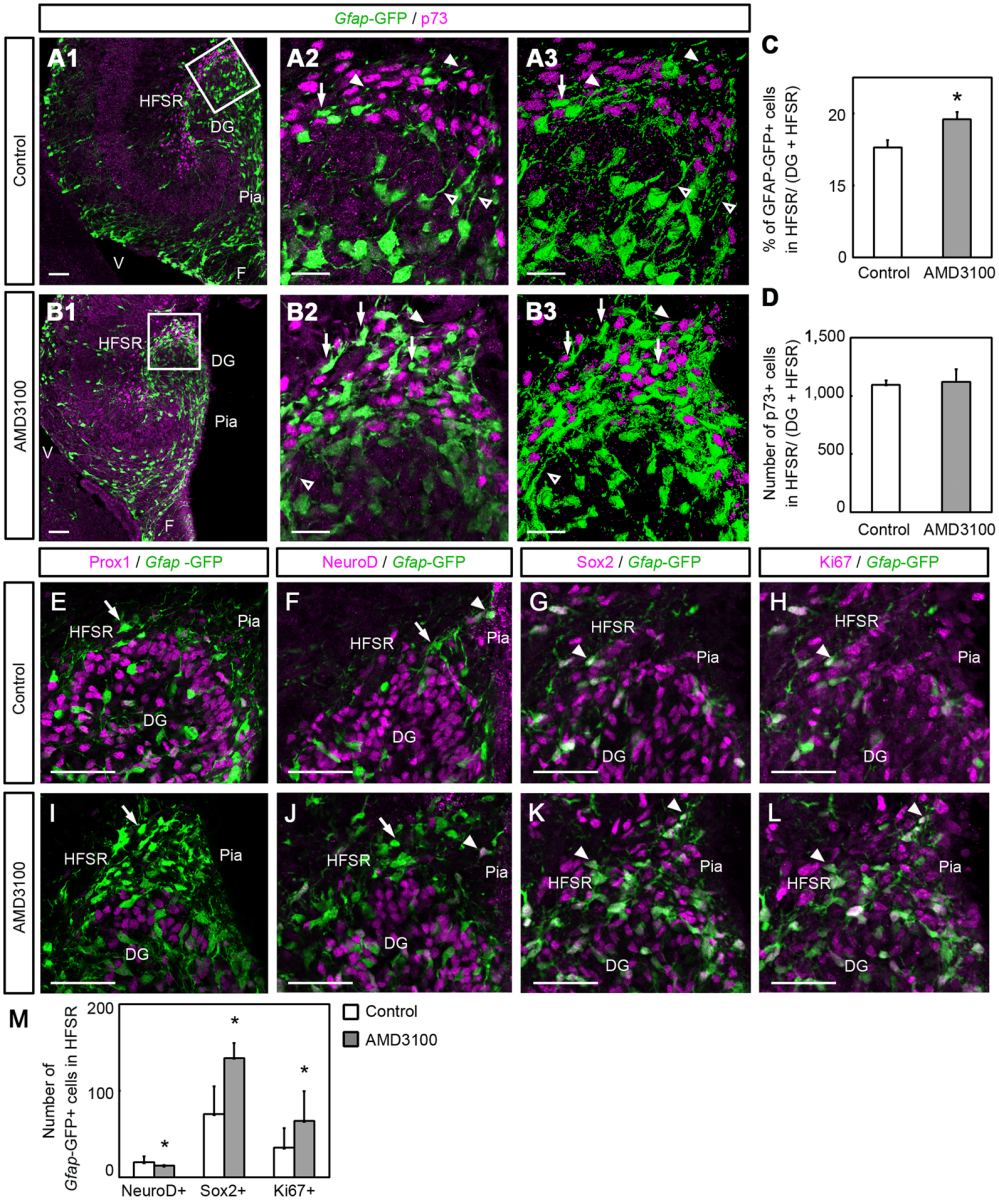
Ectopic positioning of GCPs in the hippocampal fissure-surrounding region (HFSR) induced by the CXCR4 antagonist AMD3100. AMD3100 or vehicle (control) was injected into the lateral ventricle of E15.5 embryos *in utero*, and the embryos were left to develop until E18.5. (**A,B**) Expression of *Gfap*-GFP and p73, a marker for Cajal-Retizus cells in the hippocampus of *Gfap*-GFP mice. The boxed regions in A1 and B1 are enlarged in A2 and B2, respectively. A3 and B3 are 3-dimensional images of A2 and B2, respectively, that were reconstructed from 10 optical slices. p73-positive cells accumulated in the HFSR in both the control (**A1–3**) and AMD3100-treated (**B1–3**) mice. In the control, only a few *Gfap*-GFP+ cells (arrows) were observed in the HFSR (**A1–3**), although the processes extending from the *Gfap*-GFP+ cells in the dentate gyrus (DG, open arrowheads) invaded into the HFSR (white arrowheads). In the AMD3100-injected mice, many *Gfap*-GFP-positive cells accumulated in the HFSR (**B1–3**, arrows). (**C,D**) The proportion of *Gfap-*GFP+ cells in the HFSR to those in the HFSR and DG. A significant increase in *Gfap*-GFP+ cells was observed in the AMD3100-treated mice (**C**, *P < 0.05; Student t-test). However, no difference was detected between the groups in the number of p73+ cells in the HFSR (**D**, P = 0.741, Student t-test). (**E–J**) Properties of *Gfap*-GFP-expressing cells in the HFSR. In both control and AMD3100-treated mice, *Gfap*-GFP+ cells (arrows) in the HFSR do not express the granule cell marker Prox1 (**E** and **I**), although a few GFP-positive cells express the neural progenitor marker NeuroD (arrowheads in **F** and **J**). However, the neural stem cell marker Sox2 (**G** and **K**) and the proliferation marker Ki67 (**H** and **L**) are expressed in the majority of *Gfap-*GFP-positive cells in the HFSR (arrowheads) in both groups. Results of quantitative analysis are shown in M. The number of *Gfap*-GFP+/Sox2+ cells and *Gfap*-GFP+/Ki67+ cells increased in the AMD3100-treated mice (*P < 0.05, Student t-test). We counted 10 sections from each of 3 (control) or 4 (AMD3100-treated) animals. DG, dentate gyrus; F, fimbria; V, ventricle. Scale bars = 50 µm in **A1, B1**, and **E**–**L**; 20 µm in **A2, A3, B2**, and **B3**.

As mentioned above, in AMD3100-treated embryos, CXCR4+ cells appeared to be accumulated in a region surrounding the hippocampal fissure that was occupied with p73+ cells (Fig. 7). In the present study, we call this area the hippocampal fissure-surrounding region (HFSR). To investigate the properties of the accumulated cells, we first examined the localization of *Gfap*-GFP+ cells of control and AMD3100-treated *Gfap*-GFP mouse embryos. In the control embryos, a few *Gfap*-GFP+ cells were present in the HFSR (Fig. 7A2 and A3) and the processes from *Gfap*-GFP+ cells in the DG invaded into the HFSR (Fig. 7A2 and A3). In the AMD3100-injected mice, many ectopic *Gfap*-GFP+ cells accumulated in the HFSR (Fig. 7B2 and B3). The proportion of *Gfap*-GFP+ cells in the HFSR to total GFP+ cells in the DG and HFSR significantly increased (P < 0.05, Student t-test) in the AMD3100-treated mice (19.2% ± 1.7%, n = 4, 10 sections from each of 4 embryos) compared with control mice (15.3% ± 2.3%, n = 3, 10 sections from each of 3 embryos; Fig. 7C), although no difference was detected in the number of p73-positive cells in the HFSR between the mice (Fig. 7D; control, 1,093 ± 38 cells vs. AMD3100, 1,120 ± 107 cells, P = 0.741, n = 3 and 4, 10 sections from each of 3 or 4 embryos, respectively).

Next, we analyzed the properties of *Gfap*-GFP+ cells in the HFSR using antibodies for Prox1, NeuroD, Sox2 and Ki67. In both the control (Fig. 7E,F) and AMD3100-treated mice (Fig. 7I,J), *Gfap*-GFP+ cells in the HFSR did not express Prox1, and very few GFP+ cells expressed NeuroD. In contrast, the majority of *Gfap*-GFP+ cells expressed Sox2 and Ki67 in the HFSR in both groups (Fig. 7G,H,K, and L). The number of NeuroD+/*Gfap*-GFP+ double-positive cells in the HFSR was decreased (P < 0.05, Student t-test) in the AMD3100-treated mice (13 ± 1 cells, n = 4, 10 sections from each of 4 embryos) compared with control mice (17 ± 7 cells, n = 3, 10 sections from each of 3 embryos; Fig. 7M). On the other hand, the number of Sox2+/GFP+ double-positive cells was increased *(P* < 0.05, Student t-test) in the AMD3100 treated-mice (134 ± 17 cells, n = 4, 10 sections from each of 4 embryos) compared with control mice (73 ± 31 cells, n = 3, 10 sections from each of 3 embryos; Fig. 7M). The number of Ki67+/GFP+ double-positive cells was also increased (*P* < 0.05, Student t-test) in the AMD3100 treated mice (65 ± 34 cells, n = 4, 10 sections from each of 4 embryos) compared with control mice (34 ± 23 cells, n = 3, 10 sections from each of 3 embryos; Fig. 7M). These results indicated that blocking of CXCL12/CXCR4 signaling causes the ectopic accumulation of neural progenitor-like cells in the HFSR. This suggests that CXCL12/CXCR4 signaling may regulate the final positioning of GFAP-expressing GCPs.

## Discussion

Previous studies using CXCR4-deficient or CXCL12-deficient mice indicated that CXCL12/CXCR4 signaling regulates the differentiation and migration of GCPs, as well as organization of the GCL^14, 19, 20^. However, the dynamics of the CXCR4 protein during GCP development have remained unclear. Our present study using a combination of an antibody specific for non-phosphorylated CXCR4, a λPP and an inhibitor of CXCL12/CXCR4 signaling, AMD3100 suggested that CXCL12/CXCR4 signaling induces the phosphorylation and internalization of CXCR4 as well as the intracellular punctate accumulation near the centrosomes, Golgi apparatus and lysosomes. Furthermore, functionally, this signal acts to maintain the stem cell-like properties of the GCPs, and regulates the differentiation, migration and final positioning of the GCPs. Because in the immune system and other organs, the dynamics of CXCR4 induced by CXCL12 signaling is involved in the function of CXCR4^28, 39–42^, it is probable that the phosphorylation and internalization of CXCR4 in GCPs correlate with the differentiation and migration of GCPs.

We found that in the VZ, CXCR4 is mainly present on the plasma membrane, in either the phosphorylated or non-phosphorylated form. Additionally, the number of cells with CXCR4+ intracellular puncta was low. It is well known that the binding of CXCL12 to CXCR4 activates two processes: one is a number of signaling cascades mediated via heterotrimeric G-proteins that lead to proliferation and migration, and the other is phosphorylation-dependent endocytosis of the CXCL12/CXCR4 complex followed by lysosomal degradation and desensitization of the receptor signal^24, 43^. In our present study, the existence of non-phosphorylated CXCR4 on the plasma membrane and a small number of intracellular CXCR4+ puncta imply that CXCR4 is largely available on the plasma membrane, and is not significantly down regulated. Furthermore, inhibition of CXCL12/CXCR4 signaling by the CXCR4 antagonist, AMD3100 caused a decrease in the number of proliferating progenitor cells and an increase in the number of differentiated neuronal precursors. These results suggest the precocious maturation of GCPs in the VZ. A similar type of precocious differentiation of progenitor cells has been reported in the developing cerebellum. Analysis using CXCR4-null mice showed that CXCL12/CXCR4 signaling is required for anchoring progenitors to granule cells within the external granule layer (EGL), which possesses an environment that maintains progenitor proliferation. The loss of CXCR4 leads to the premature migration of the progenitors away from the EGL^44, 45^. Taken together, it is most likely that CXCR4-mediated signaling functions in maintaining stem cell-like features of the GCPs in the VZ and preventing their precocious differentiation.

In the DMS, CXCR4 was found both on the plasma membrane and in the intracellular puncta, the latter of which were present close to the centrosome, Golgi apparatus and lysosomes. CXCR4 molecules in the plasma membrane were primarily phosphorylated. When CXCL12/CXCR4 signaling was inhibited, most CXCR4 molecules were confined to the plasma membrane and were dephosphorylated. These results suggest that the formation of CXCR4+ intracellular puncta and phosphorylation of CXCR4 are regulated by CXCL12/CXCR4 signaling. In the immune system, phosphorylation-dependent internalization of CXCR4 is well documented. CXCR4 on the plasma membrane of T-cells, after exposure to CXCL12, is rapidly internalized, and then trafficked to the Golgi apparatus to regulate T-cell migration^41^, or is sorted to lysosomes^46^ to be degraded or recycled^39–41^. It has been proposed that the internalization of CXCR4 receptors from the cell surface and their degradation by lysosomes cause a decrease in the number of CXCR4 on the cell surface, and regulate the availability of CXCR4 receptors, as well as the magnitude and duration of signaling. Studies of hematopoietic stem cells indicate that a high level of CXCL12 induces desensitization and receptor internalization, whereas a low level of CXCL12 promotes cell motility, proliferation, and migration^47–49^. In the adult hippocampus and in primary embryonic hippocampal culture, the level of CXCR4 in CXCL12-stimulated neurons is reported to be under dual control by the recycling and degradation of internalized CXCR4^50^. Taken together, it is probable that the binding of CXCL12 to CXCR4 on the plasma membrane of the GCPs induces the phosphorylation and internalization of CXCR4, and the internalized CXCR4 is sorted to the centrosome, Golgi apparatus, and lysosomes.

A few studies have reported the intracellular punctate expression of CXCR4 in the developing nervous system, but the function of these CXCR4+ punctate structures remains unclear. In cultured neuronal progenitors from the E15 rat brain, CXCR4 was found as punctate aggregates in the perinuclear region^51^. In developing CXCR7-null mice in which CXCL12 levels are increased, intracellular clustering of CXCR4 is detected in migrating GnRH-positive neurons, although similar clusters are not found in wild-type mice^52^. This implies that excess CXCL12/CXCR4 signaling causes the intracellular clustering of CXCR4. In the adult hippocampus, CXCR4 expression is found in the plasma membrane and intracellular puncta of neural progenitors/precursors in the subgranular zone, and inhibition of CXCL12/CXCR4 signaling leads to an increase in the number of cells with a CXCR4+ membrane^50^. In addition to the intracellular punctate expression of CXCR4, we further found the association of CXCR4+ puncta with the centrosome, Golgi apparatus, and lysosomes. As mentioned above, CXCR4 that is sorted to lysosomes may be degraded or recycled. However, the roles played by CXCR4 that is trafficked to the centrosome and Golgi apparatus remain unclear. In this respect, it has been reported that the centrosome and Golgi apparatus are associated with cell migration^41, 53, 54^. It is hence possible that CXCR4 in association with the centrosome and Golgi apparatus play a role in the migration of GCPs.

In the present study, we found that the inhibition of CXCL12/CXCR4 signaling causes a reduction in the number of Prox1+ cells in the DG, suggesting the delayed migration of GCPs. Furthermore, in this condition, the GCPs underwent precocious differentiation before arriving at the DG. Similar delayed migration has been shown by studies using CXCR4-deficient and CXCL12-deficient mice. These studies indicated that CXCL12/CXCR4 signaling regulates the migration of CR cells^55, 56^, GnRH neurons in the forebrain^52^, and dopamine neurons in the midbrain^57^. In the hippocampus of CXCR4-deficient mice, the delayed migration and premature differentiation of GCPs has been reported^14, 19, 20^. Taken together, these studies suggest that in the DMS, CXCR4 is involved in the migration and differentiation of GCPs.

Normally, GCPs migrate and settle in the DG where they differentiate into granule cells or continue to proliferate to produce granule cells. However, in AMD3100-treated mice, many GCPs were ectopically detected in the HFSR, suggesting that inhibition of CXCL12/CXCR4 signaling disrupts the final positioning. It is not clear how GCPs normally stop and settle in the DG, and how the inhibitor disturbs the positioning. In this respect, in zebrafish, the internalization of CXCR4 was reported to be essential for the precise arrival of primordial germ cells at the target in CXCL12-guided cell migration^58^. Consistently, we found that in the DG, CXCR4 was mainly present in intracellular punctate structures. Thus, one possibility is that the internalization of CXCR4 in GCPs is involved in their precise positioning within the DG, and the inhibition of internalization disrupts this positioning. In the immune system, it has also been reported that immune cells are attracted by low concentrations of CXCL12, but are repulsed by high concentrations of CXCL12^59^. In the developing hippocampus, the concentration of CXCL12 is thought to be very high in the HFSR, because the HFSR contains many CR cells that secrete CXCL12. Therefore, another possibility is that the high CXCL12 concentration serves as a repellent to the GCPs, and prevents invasion of the GCPs into the HFSR.

The intracellular distribution pattern of CXCR4 and the AMD3100-induced alteration in the GCPs depended on the hippocampal region, such as the VZ, DMS, and DG. In the hippocampus, CXCL12 is secreted by CR cells located in the HFSR. Concentrations of the CXCL12 signal may decrease from the DG to the VZ. CXCL12 stimulation is well known to cause the phosphorylation and subsequent internalization of CXCR4. The concentration of CXCL12 could define the extent of phosphorylation and internalization of CXCR4. During the migration of GCPs from the VZ to DG via the DMS, CXCR4 can be gradually phosphorylated in response to CXCL12, and then progressively internalized and accumulated as the GCPs approach the DG. It is thus possible that the different dynamics and functions of CXCR4 within these three regions are induced by the different intensity of CXCL12 signals.

**Figure 8:**
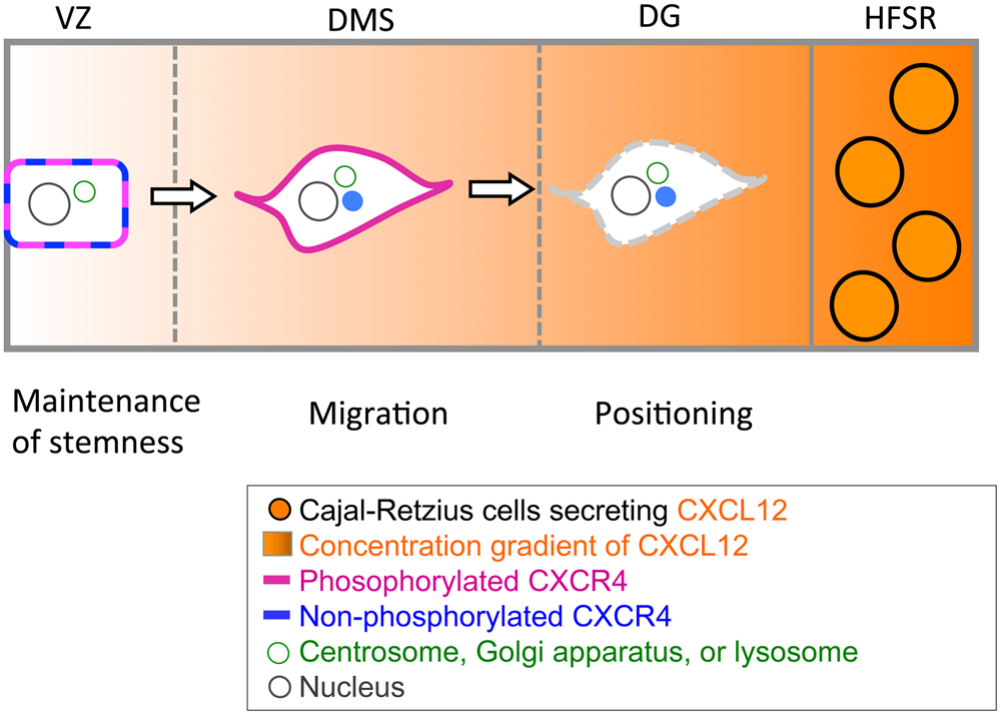
A hypothetical model of the dynamics and function of CXCR4 in the formation of the GCL during hippocampal development. CXCL12 is secreted by Cajal-Retzius cells located in the hippocampal fissure-surrounding region (HFSR). A gradient of CXCL12 molecule concentration is thought to exist in the hippocampus, decreasing from the DG to the VZ. CXCL12 stimulation is well known to cause the phosphorylation and subsequent internalization of CXCR4. Thus, the proposed hypothesis is as follows. In the VZ (low level of CXCL12), CXCR4 is mainly present on the plasma membrane, and is partially phosphorylated. CXCR4 signaling may function in maintaining the stemness of ventricular cells. In the DMS (medium level of CXCL12), CXCR4 on the plasma membrane is mostly phosphorylated and partially internalized. The internalized CXCR4 traffics to the centrosomes, Golgi apparatus or lysosomes and is dephosphorylated where it may play a role in cell migration. In the DG (high level of CXCL12), CXCR4 is mostly internalized, and accumulates as intracellular punctate structures close to the centrosomes, Golgi apparatus, or lysosomes, and also becomes dephosphorylated. CXCR4 may thus regulate the final positioning of granule cell progenitors.

On the basis of this concept, we propose a hypothetical model for the dynamics and function of CXCR4 in the formation of the GCL during hippocampal development (Fig. 8). In the VZ, which is located far from the HFSR, CXCL12 levels are very low, which induces the partial phosphorylation of CXCR4. In this situation, the amount of internalized CXCR4 is very low. As a result, CXCR4 is mainly present on the plasma membrane, in either its phosphorylated or non-phosphorylated form. When CXCR4 is located on the plasma membrane of cells in the VZ, CXCR4 may be involved in maintaining stem cell-like features of the GCPs in the VZ, regulating neural differentiation, and preventing precocious maturation. In the DMS, which is the mid-point between the VZ and DG, the CXCL12 levels are moderate. The moderate levels of CXCL12/CXCR4 signaling result in the complete phosphorylation of CXCR4 and the partial internalization of CXCR4. The internalized CXCR4 accumulates as punctate aggregates close to the centrosomes, Golgi apparatus, and lysosomes. This internalized CXCR4 can be recycled or degraded when it is sorted to the lysosomes, or might function in cell migration with microtubules when it traffics to the centrosomes and Golgi apparatus. In the DG near the HFSR, CXCL12 levels are high, and CXCR4 is mostly internalized, and accumulates as intracellular punctate structures close to the centrosomes, Golgi apparatus, and lysosomes. This condition may regulate the final positioning of the GCPs. This hypothesis provides new insight into the association between the dynamics and function of morphogens and their receptors in neural development.

## Methods

### Animals

The generation of *mouse* G*fap*-GFP mice^60^ has been described previously. Mice were maintained under standard conditions (12-hour light/dark cycle) in the animal care center of Tokyo Medical University. All experiments were performed in accordance with institutional, science community, and national guidelines for animal experimentation and approved by the Institutional Animal Care and Use Committee (IACUC) of Tokyo Medical University and also conform to the National Institutes of Health Guide for the Care and Use of Laboratory Animals (NIH publication no. 80-23), revised in 1996. All efforts were made to minimize the number of animals used and their suffering. For timed mating, the day of vaginal plug detection was counted as embryonic day 0.5 (E0.5). The day of birth was counted as postnatal day 0. Forty-two embryos were used in this study.

### *In utero* electroporation and intraventricular injection

*In utero* electroporation of mouse embryos was performed as previously described^61^ with minor modifications^61^. Briefly, pregnant wild-type mice at E15.5 were anesthetized with sodium pentobarbital. After cleaning the abdomen with 70% ethanol, a midline incision of approximately 3 cm was made. The uterus was exposed, and the lateral ventricle of the embryos was identified under transillumination. The *pCAGGS-*BFP plasmid constructed from a vector developed by Niwa *et al*.^62^ was diluted with PBS (final DNA concentration: 0.5 µg/ml), and 0.5 µL of the solution was injected into the lateral ventricle with a glass capillary pipette connected to an electric injector (BJ-110, BEX Co. Ltd.). Square pulses (30 V, 50 milliseconds, 4 times at 1-second intervals) were delivered to the neocortex with tweezer-type electrodes, which consisted of a pair of round platinum plates that were 3 mm in diameter (LF650P3, BEX, Tokyo, Japan), and an electroporator (CUY21, Nepa Gene, Chiba, Japan). The electroporated embryos were subsequently fixed at E18.5.

For the blocking of CXCL12/CXCR4 signaling, surgery was performed in the same manner as described above. One µL of AMD3100 solution (12.6 mM in PBS; Sigma-Aldrich, St. Lous, MO) or PBS was injected into the telencephalic lateral ventricle of E15.5 embryos *in utero*, and embryos were left to develop until E18.5.

### Tissue preparation

For embryonic time points, pregnant mice were deeply anesthetized with sodium pentobarbital and embryos were removed via laparotomy. The brains were removed from the skull and immersion-fixed overnight in 4% paraformaldehyde in 0.1 M PB at 4 °C. The fixed brains were washed with PBS and the forebrains were embedded in OCT compound and stored at −80 °C. The forebrains were sectioned serially and coronally into 20-µm slices using a cryostat (Fig. 1), or the medial cerebral cortices including the hippocampus were cut serially and perpendicularly to the septo-temporal axis in the same manner (Figs 2–7). Every four sections were mounted on slides and were subjected to immunohistochemistry and quantification.

### Immnohistochemistry

Primary and secondary antibodies were diluted with 1% Triton X-100 in PBS (PBS-T) containing 1% bovine serum albumin. For triple staining, sections were incubated with a mixture of primary antibodies at 4 °C for 1 to 2 nights and then with a mixture of secondary antibodies and Hoechst 33275 at room temperature for 1 to 2 hours. Each step was followed by washing with PBS-T. The slides were covered with IMMU-MOUNT (Thermo Fisher Scientific Inc.). The primary antibodies used were as follows: anti-CXCR4 UMB2 (1:200, rabbit monoclonal, Abcam), anti-GFP (1:1,000, chick polyclonal, Abcam), anti-GM130 (1:500, mouse monoclonal, BD Transduction Laboratories), anti-γ-tubulin (1:200, mouse monoclonal, Sigma), anti-Ki67 (1:500, rabbit polyclonal, Novocastra), anti-LAMP1 (1:1000, rat monoclonal, Abcam), anti-NeuroD (1:200, goat polyclonal, Santa Cruz Biotechnology, Inc.), anti-Prox1 (1:1,000, goat polyclonal, R&D Systems), anti-p73 (1:200, rabbit polyclonal, Santa Cruz Biotechnology, Inc.). Secondary antibodies used were as follows: Alexa Fluor 488-conjugated anti-chicken IgG, Alexa Fluor 546-conjugated anti-rabbit IgG, Alexa Fluor 647-conjugated anti-goat IgG (donkey polyclonal 1:500, Invitrogen, Carlsbad, CA), Cy2-conjugated anti-mouse IgG, and Cy5-conjugated anti-rat IgG (1:200, donkey, Jackson ImmunoResearch Laboratories, West Grove, PA). The sections were examined with a Zeiss confocal laser-scanning microscope 700 with 20x and 63x objectives and in some cases fluorescence images were digitally zoomed at 0.5x to 2x. Stacks of optical sections (0.9–1.8 µm in thickness) were obtained at 0.45-µm and 0.9-µm increments on the z-axis, respectively. When the primary antibodies were omitted from the immunohistochemical staining, no immunopositive staining was observed except for those using anti-mouse IgG secondary antibodies. In the case of the anti-mouse IgG antibodies, some blood vessels were non-specifically stained, but they were easily distinguished from the specific staining, and were not counted in quantitative analyses. Tiling images were produced with ZEN processing software (Carl Zeiss, Oberkochen, Germany). Images were analyzed using Zeiss LSM700 software or ImageJ.

To detect total CXCR4 (both phosphorylated and non-phosphorylated forms), sections of the hippocampus were treated with λPP according to the method previously described by Yang *et al* ^57^, because anti-CXCR4 UMB2 specifically recognizes non-phosphorylated CXCR4^28, 32^. Adjacent sections were incubated with λPP (800 U; New England Biolabs) in Protein MetalloPhosphatases buffer with MnCl_2_ or with the same buffer for 1 hour at room temperature. Immunostaining for CXCR4 in adjacent sections was performed simultaneously in the same condition. The intensity of CXCR4 immunoreacitivity was measured using imageJ.

### Immunoelectron microscopy

The forebrains were fixed, embedded as described above and sectioned into 50-µm slices using a cryostat. The sections were immunostained with anti-CXCR4 UMB2 (1:200, rabbit monoclonal, Abcam), visualized with a 3,3″-diaminobenzidine tetrahydrochloride reaction and embedded in Epon. Ultrathin sections were examined by JOEL 1200 (JOEL, Tokyo, Japan).

### Statistical analysis

All data are reported as the mean ± S.D.; analysis for statistical significance was assessed by the two-tailed paired Student’s *t*-test (Figs 5–7, Supplemental Fig. 4) or one-way ANOVA and Tukey-Kramer test (Supplemental Fig. 3).

## Electronic supplementary material

Supplemental Information
