# Supplementary material for: Dynamics and function of CXCR4 in formation of the granule cell layer during hippocampal development
Source: Sci Rep. 2017 Jul 17;7:5647. doi: 10.1038/s41598-017-05738-7 (PMC5514042; doi:10.1038/s41598-017-05738-7)
Supplement: Supplementary file 1 — Supplemental Information [file 41598_2017_5738_MOESM1_ESM.doc]

**Dynamics and function of CXCR4 in formation of the granule cell layer during hippocampal development**

Yuka Mimura-Yamamoto^1^, Hiroshi Shinohara^1^, Taichi Kashiwagi^1^, Toru Sato^1^, Seiji Shioda^2^ and Tatsunori Seki^1,*^

^1^Department of Histology and Neuroanatomy, Tokyo Medical University, Tokyo, 160-8402, Japan

^2^Institute for advanced Bioscience Research, Hoshi University, Tokyo, 142-8501, Japan

**Corresponding author:**

Tatsunori Seki, Ph.D.

Department of Histology and Neuroanatomy

Tokyo Medical University

6-1-1 Shinjuku, Shinjuku-ku, Tokyo 160-8402, Japan

Phone: +81-(0)3-3351-6141 ext. 274

Fax: +81-(0)3-3351-7886

Email: [sekit@tokyo-med.ac.jp](mailto:sekit@tokyo-med.ac.jp)


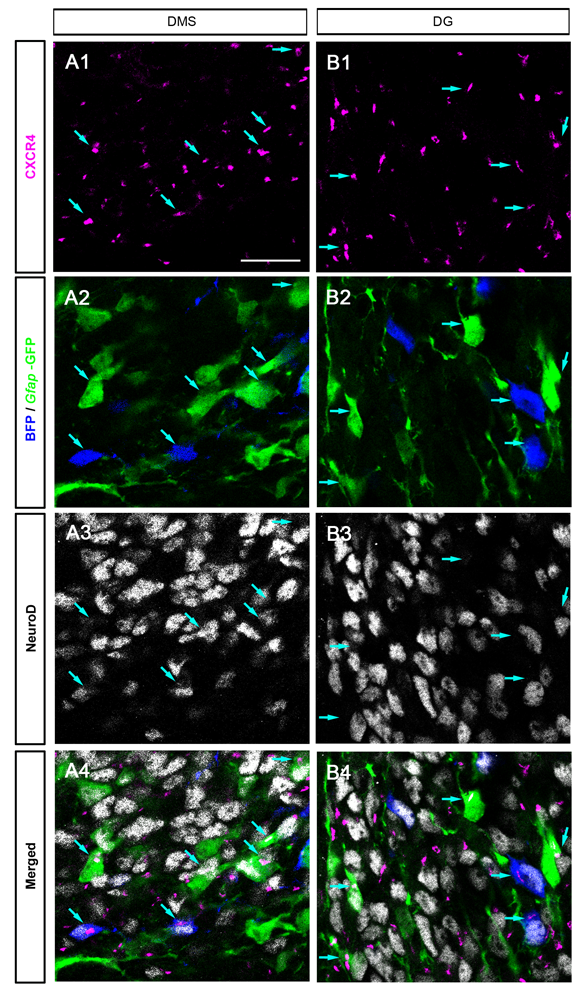


Supplemental Figure 1

CXCR4 accumulated as puncta in migrating neuronal precursor cells. The *pCAGGS-*BFP plasmid was electroporated into the ventricular surface of the hippocampal region of *Gfap*-GFP mouse embryos at E15.5, and they were subsequently fixed at E18.5. CXCR4+ puncta (blue arrows) were observed not only in *Gfap*-GFP+ cells, but also in BFP+ cells with a NeuroD+ nucleus in the dentate migratory stream (DMS) and dentate gyrus (DG). Scale bar = 20µm in A1-B4.


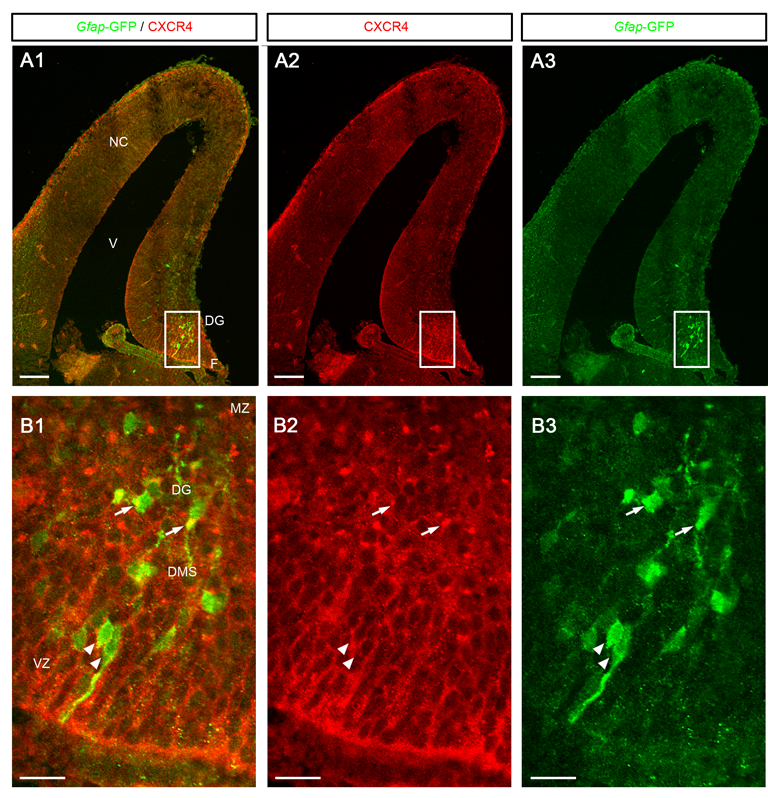


Supplemental Figure 2. Expression of CXCR4 and *Gfap*-GFP in the cerebral hemisphere, including the neocortex and hippocampus at E14.5. The boxed regions in A1, A2, and A3 are enlarged in B1, B2, and B2, respectively. In the ventricular zone (VZ), CXCR4 is expressed on the plasma membrane of *Gfap*-GFP cells (arrowheads). In the dentate migratory stream (DMS) and prospective dentate gyrus (DG), CXCR4+ puncta (arrows) are found in the *Gfap*-GFP cells. F, fimbria; NC, neocortex; V, ventricle. Scale bars = 100 µm in A1-3; 20 µm in B1-B3.


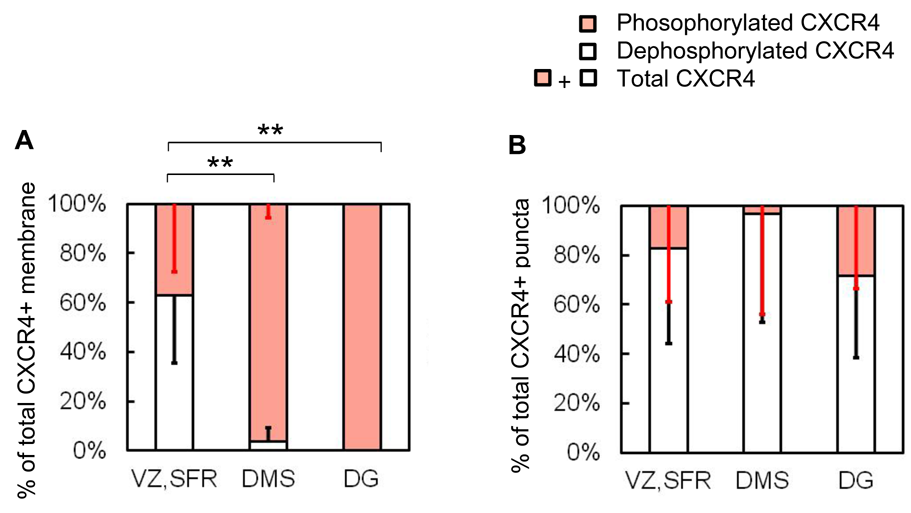


Supplemental Figure 3. Percentages of cells with phosphorylated or dephosphorylated CXCR4+ plasma membranes to cells with total (phosphorylated and dephosphorylated) CXCR4+ plasma membranes (A), and percentages of cells with phosphorylated or dephosphorylated CXCR4+ intracellular puncta to cells with total CXCR4+ intracellular puncta (B). The percentage of cells with dephosphorylated CXCR4+ plasma membrane is decreased in the VZ-suprafimbrial region (SFR) and DG, whereas the percentage of cells with phosphorylated CXCR4+ plasma membrane is increased in the VZ and DG (A). On the other hand, there is no difference in the percentage of cells with phosphorylated and dephosphorylated CXCR4+ puncta among the VZ-SFR, DMS and DG (B).


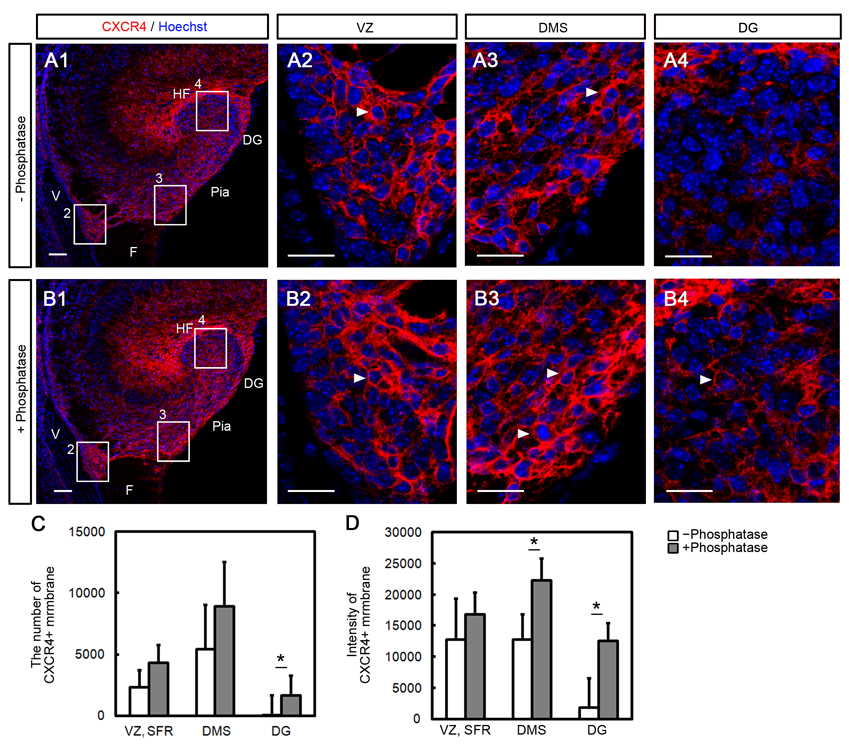


Supplemental Figure 4

Non-phosphorylated (A) and total (B) CXCR4 expression in the hippocampus of AMD3100-treated embryos at E18.5. Non-phosphorylated CXCR4 was detected by the UMB2 antibody, which specifically recognizes the non-phosphorylated C-terminal domain of CXCR4. To detect total CXCR4 (both the phosphorylated and non-phosphorylated forms), hippocampal sections were treated with lambda protein phosphatase (λPP). Immunostaining with or without λPP in adjacent sections was performed simultaneously in the same condition. (A1-4) Detection of non-phosphorylated CXCR4. The boxed regions in A1 are enlarged in A2, A3 and A4, as indicated. In the ventricular zone (VZ) and dentate migratory stream (DMS), CXCR4 is present in the plasma membrane (arrowheads in A2, A3, B2 and B3). In the dentate gyrus (DG), almost no CXCR4 is found on the plasma membranes in a non-phosphatase-treated section, whereas weak CXCR4 expression is detected in a phosphatase-treated section (B4, arrowhead). Quantitative analysis of the number of cells with CXCR4 on their plasma membranes, and immunoreactive intensities of CXCR4 in the membrane are shown in C and D, respectively. Dephosphorylation by phosphatase did not significantly alter the number of cells with CXCR4 on their plasma membrane in the VZ-suprafimbrial region (SFR) (-PP, 2,290 ± 2,384 cells vs. +PP, 4,320 ± 1,428 cells, P =0.134, n = 6, 6 sections in 6 embryos, Student t-test) and DMS (-PP, 5,408 ± 2,706 cells vs. +PP 8,912 ± 3,604 cells, P =0.113, n = 6, 6 sections in 6 embryos, Student t-test), suggesting that the majority of migrating cells have dephosphorylated CXCR4 in AMD3100-treated embryos, probably because of the absence of CXCL12 stimulation. The intensity of CXCR4 on the plasma membrane is increased in the DMS (-PP, 12,781 ± 4,049 vs. +PP, 22,239 ± 3,545, P < 0.05, n = 6, 6 sections in 6 embryos, Student t-test) and DG (-PP, 1,800 ± 4,697 vs. +PP, 12,493 ± 2,877, P < 0.05, n = 6, 6 sections in 6 embryos, Student t-test). This may be due to the persistence of phosphorylated CXCR4, existing from before AMD3100 treatment, because internalization of CXCR4 is inhibited by AMD3100. F, fimbria; HF, hippocampal fissure; V, ventricle. Scale bars = 50 µm in A1, B1; 20 µm in A2-4, B2-4.
